# Treatment of a Case of Disease in the Antrum of Highmore

**Published:** 1893-04

**Authors:** J. B. Van Fossen


### THE DENTAL REGISTER.

VOL. XLVII.] APRIL, 1893. [No. 4.

### COMMUNICATIONS.

### Treatment of a Case of Disease in the Antrum of Highmore.

BY J. B. VAN FOSSEN, D.D.S.

Read before the Detroit Dental Society, March 13th, 1893.

This curious opening is located in the body of the superior maxillary bone. There are several theories in regard to its real office. Some claim it is to make the bones of the face a little lighter; others, that it is a kind of sound apparatus to give pitch to the voice; and still others, who claim the distribution of the olfactory nerve over its surface which is lined with mucous membrane aids in the sense of smell. However this may be, there is one thing which it does, and that is to make the disease it is subject to very disagreeable for the dentist as well as for the patient. The diseases which are caused directly by the teeth are the ones that the dentist is most interested in.

This curious space has but one opening leading to the nose. The roots of the teeth very often puncture the floor of the antrum and in case of ulceration are very apt to make a disturbance.

The case to which I wish to call your attention, was one I had in practice but a few months ago. The patient, a lady, about forty-five years of age, I should judge. The first time I saw her was during the summer, and at that time she complained of a dull, heavy pain on the right side of her face, just below the eye. There seemed to be a slight swelling at this time. I examined her mouth and advised her to have all the teeth in the upper jaw removed. She consented, and I at that time extracted them all. The tooth that seemed to have made the trouble, was the first superior molar, and I remember I had some difficulty in taking it out. The tooth was free from decay, and the only one that was in a good state of preservation. I did not see the patient for about six weeks, when she called upon me again. At this time her face was badly swollen, and she informed me that she had been confined to her bed the most of her time since I removed her teeth; her physician treating her for I don't know how many things. Upon examining her mouth, I found the opening where I had removed the molar tooth, had never started to heal, and there was a small tit-like substance hanging from the opening, resembling gristle in structure. I took a small pair of pliers and with some difficulty removed it; there did not seem to be any discharge at that time from the artificial opening. After removing this substance, I, at once, mistrusted what the matter was, but, as I had nothing to treat it with, I plugged up the opening with a piece of cotton rolled hard and moistened with oil of eucalyptus, I then dismissed the patient, requesting her to call the next day. On her return I took one of the largest sized "rose burs" and drilled through until I struck the floor of the antrum—making a good free opening. I then had the patient bend well forward over a large wash-bowl and with a small rubber syringe I washed the cavity out thoroughly using as much force as I could in throwing the water into the antrum. I soon discovered I had a good large opening, as the most of the discharge came through the nose. The discharge was at first quite black, but free from odor. After washing it out with the warm water, I again washed it out with peroxide of hydrogen and then with a four per cent solution of boracic acid. After using the peroxide the patient would complain of a stinging sensation for some time, and, I think, had I not had such a good opening it would have been better not to have used so much of it. Before dismissing the patient I was always careful to cork the opening as tight as I could to keep the secretions of the mouth out. I followed this treatment up every day for about a week without seeing any change to amount to anything. I then increased it to three times a day, and followed this up for about two weeks; at the end of that time I could scarcely detect any discoloration in the discharge whatever. I then treated it once a day for about a week, and then about twice a week. It was but a short time before it commenced to heal—all pain subsided—swelling disappeared and the patient got well.

In conclusion I would like to ask the members of the society if they think, I in any way could have caused this disturbance in the extraction of the tooth.

